# Acceptance of COVID-19 vaccine in Pakistan among health care workers

**DOI:** 10.1371/journal.pone.0257237

**Published:** 2021-09-15

**Authors:** Asmara Malik, Jahanzeb Malik, Uzma Ishaq

**Affiliations:** 1 Department of Community Medicine, Army Medical College, National University of Medical Sciences, Rawalpindi, Pakistan; 2 Department of Cardiology, Rawalpindi Institute of Cardiology, Rawalpindi, Pakistan; 3 Department of Hematology, Foundation University Medical College, Islamabad, Pakistan; Health Services Academy Islamabad Pakistan, PAKISTAN

## Abstract

**Objective:**

Acceptance of the COVID-19 vaccine will impart a pivotal role in eradicating the virus. In Pakistan, health care workers (HCWs) are the first group to receive vaccination. This survey aimed at the level of acceptance to the COVID-19 vaccine and predictors of non-acceptance in HCWs.

**Method:**

This was a cross-sectional study design and data were collected through 3rd December 2020 and February 14th, 2021. An English questionnaire was distributed through social media platforms and administration of affiliate hospitals along with snowball sampling for private hospitals.

**Results:**

Out of 5,237 responses, 3,679 (70.2%) accepted COVID-19 vaccination and 1,284 (24.5%) wanted to delay until more data was available. Only 5.2% of HCWs rejected being vaccinated. Vaccine acceptance was more in young (76%) and female gender (63.3%) who worked in a tertiary care hospital (51.2%) and were direct patient care providers (61.3%). The reason for rejection in females was doubtful vaccine effectiveness (31.48%) while males rejected due to prior COVID-19 exposure (42.19%) and side effect profile of the vaccine (33.17%). Logistic regression analysis demonstrated age between 51–60 years, female gender, Pashtuns, those working in the specialty of medicine and allied, taking direct care of COVID-19 patients, higher education, and prior COVID-19 infection as the predictors for acceptance or rejection of COVID-19 vaccine.

**Conclusion:**

In conclusion, this survey suggests that early on in a vaccination drive, majority of the HCWs in Pakistan are willing to be vaccinated and only a small number of participants would actually reject being vaccinated.

## Introduction

The novel coronavirus disease 2019 (COVID-19) was declared as a pandemic and an emergency was initiated by World Health Organization (WHO) on 30th January 2020 [1]. The outbreak revealed itself as clusters of pneumonia of unknown etiology in China [2]. A systematic review has outlined a severe form of the disease in 20% of the affected individuals with a mortality rate of 3% [3]. As of February 2021, COVID-19 has affected 108 million people worldwide, leading to 2.38 million deaths [4] while Pakistan has reported 560,000 cases and 12,218 deaths [5]. Hence, in addition to social distancing measures and personal protective equipment [6], there is a vital need to be vaccinated for COVID-19 to curb the community transmission in Pakistan.

Health care workers (HCWs) have an important part in educating the general public about the source of the vaccine and its implications in the coming years [7, 8]. In Pakistan, HCWs are being prioritized for an early Chinese-based COVID-19 vaccination program [9]. This is being mandated throughout the West, prioritizing high-risk groups, and HCWs being recognized as such. Therefore, it is vital to consider HCW attitudes towards the COVID-19 vaccine as it will lead to a better dissemination of knowledge among the general public.

Given the paucity of data regarding vaccine acceptance in South-East Asia among HCWs, we conducted this survey across multiple healthcare facilities throughout Pakistan to measure the acceptance of COVID-19 vaccine, and to enumerate the reasons underlying vaccine hesitancy among HCWs.

## Methods

### Study design and sampling

This study was a cross-sectional design to assess the acceptability of HCWs towards the COVID-19 vaccination program in Pakistan. An English questionnaire was designed on Google Forms from a previous study and modified for HCWs using data capture tools hosted at the Foundation University. No identifying information was presented in the survey and all data were collected anonymously. All rights for sharing the survey questionnaire belongs to the University. The Foundation University Ethical Review Committee approved the study design (Number: FFH/51/DCA/2020).

The questionnaire was distributed on social media platforms and a large coverage was made available by our affiliate institutes in major cities of Pakistan. Snowball sampling was also encouraged for dissemination of the survey questionnaire in primary care and private hospitals. Data were collected through 3rd December 2020 and February 14th, 2021. Informed written consent was taken before final form submission and all adults (age ≥ 18 years) working as HCW were considered eligible to participate in the survey. Incomplete questionnaires were excluded from the final analysis.

### Study variables and measures

Demographic data was presented on the first page of the survey questionnaire. It included age, gender, ethnicity, marital status, type of designated work, education, type of medical facility, chronic medical conditions, and prior COVID-19 infection. To assess the acceptance of the COVID-19 vaccine, the respondents were provided the brand name and effectiveness of the vaccine (CanSino Biologics, Tianjin, China; 65.7% effective in preventing symptomatic cases). Respondents were given a question of whether they would accept the above-labeled vaccine as yes or no. As for demographic variables, age was grouped into five categories (18–30, 31–40, 41–50, 51–60, >60 years old); ethnicity was grouped as Punjabi, Sindhi, Balochi, Pashtun, or other to find out which group was interested in vaccinating among HCWs; and designation of work was divided into direct patient care providers (Specialists, general practitioners, medical students, and nursing staff) and non-patient care providers (hospital supporting staff, administration, and pharmacists). Type of education and specialty were broken down into medicine/allied, surgery/allied, diagnostics, or other. Place of work was designated as either a tertiary care hospital, primary care center, or a private clinic.

### Statistical analysis

For analysis of the data, Statistical Package for Social Sciences (SPSS) version 26 (IBM, Armonk, NY, USA.) was used and logistic regression was employed to determine the predictors of HCWs acceptance of COVID-19 vaccine. Continuous variables are presented as mean and standard deviation (SD) and categorical variables as frequency and percentages. Student’s t test was used for continuous variables and chi-square for categorical variables. Univariate analysis was done for unadjusted estimate of odds ratio (OR) and multivariate analysis for adjusted OR. Logistic regression was carried out for predictors of vaccine hesitancy. A p-value of less than 0.05 was considered statistically significant.

## Results

### Respondent demographics

We received 5,381 responses. One-hundred and forty-four were excluded because of the incomplete survey questionnaire. A total of 5,237 responses were included in this study. More than two-thirds of the respondents were younger than 50 years (76%) and 63.3% were females who had either a bachelor’s (28.2%) or a master’s degree (11.6%). Overall, 51.2% of respondents worked in tertiary care hospitals and 61.3% were direct patient care providers. The majority of the responses were received from Punjab (43.1%) in the specialty of medicine and allied (37.9%). Sixty-three percent had a history of COVID-19 disease before this survey.

### Vaccine acceptance and predictors

**Fig 1:**
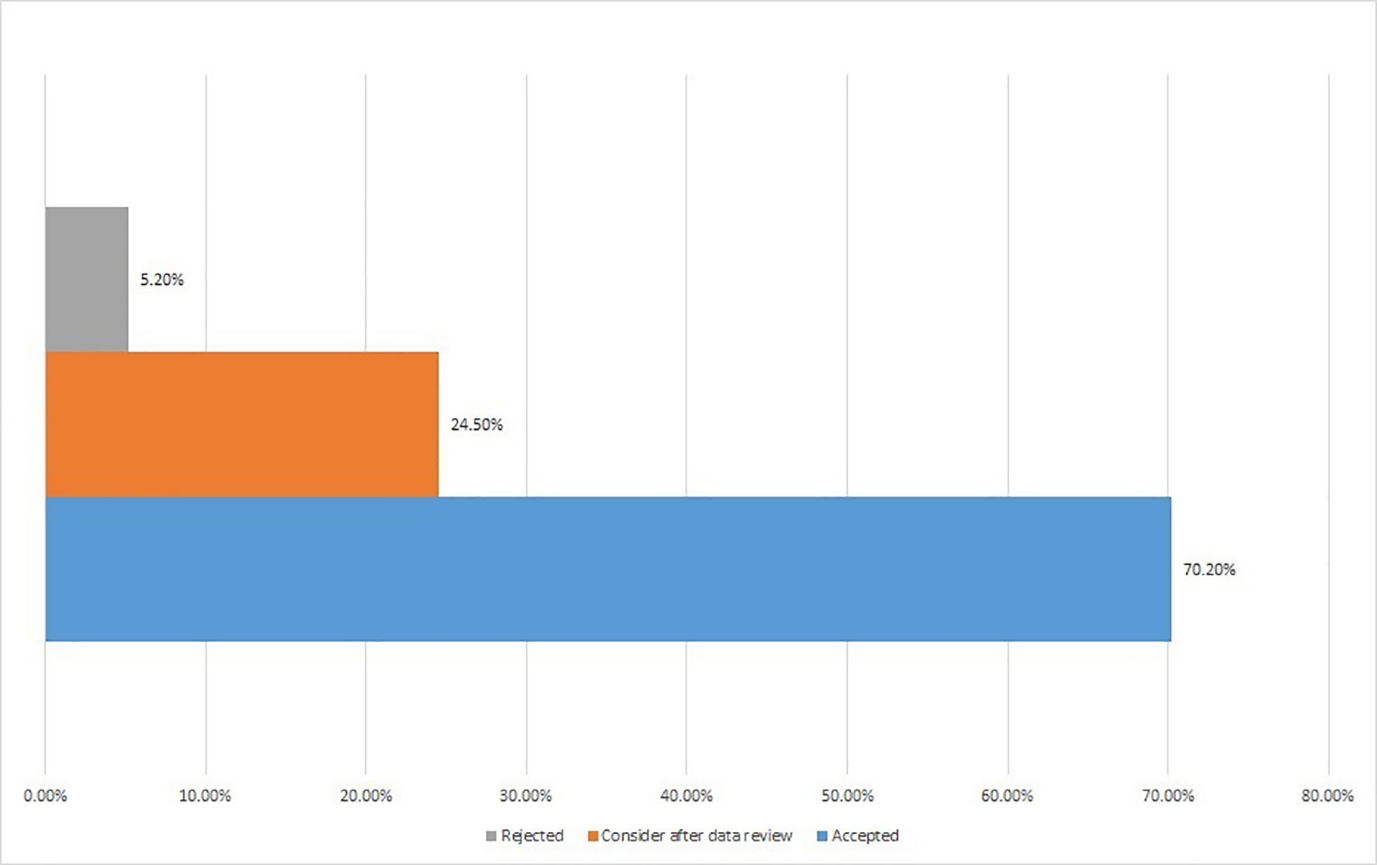
Overall acceptance, acceptance after data review, and rejection rate among HCW’s. Health care workers (HCW).

Out of 5,237 respondents, 3,679 (70.2%) accepted the vaccination process while only 274 (5.2%) rejected it. One-fourth of the HCW’s would review data on COVID-19 vaccine before moving further with the vaccine (24.5%). This is expressed in **Fig 1**. There was a significant association between vaccine acceptance and respondent demographics. Respondent demographic details, chronic medical conditions, and percent acceptance of COVID-19 vaccine are presented in **Table 1** along with age, ethnicity, and gender-adjusted response variables.

**Table 1:** Demographic data and logistic regression analysis demonstrating factors associated with acceptance of a COVID-19 vaccine in health care workers in Pakistan, n = 5,237.

Variable	n (%) 5237	Vaccine acceptance; n (%); 3679 (70.2%)	Unadjusted		Adjusted	
			OR (95% CI)	p-value	aOR (95% CI)	p-value
**Age**						
18–30	1,294(24.7%)	837 (64.6%)	0.93(0.49–1.81)	0.536	1.21(0.55–2.17)	0.707
31–40	1,912(36.5%)	1,362 (71.2%)	0.36(0.18–0.84)	0.012	0.48(0.19–1.25)	0.053
41–50	775(14.8%)	392(50.5%)	1.79(0.23–2.76)	0.465	1.93(0.27–3.01)	0.621
51–60	911(17.4%)	831(91.2%)	0.61(0.21–1.81)	0.001	0.54(0.19–1.67)	0.003*
≥60	345(6.6%)	257(74.4%)	0.72(0.36–0.91)	0.800	0.80(0.41–0.97)	0.782
**Gender**						
Male	1,922(36.7%)	1,002(52.4%)	1.45(1.01–2.58)	0.387	1.34(1.41–2.98)	0.551
Female	3,315(63.3%)	2,678(80.7%)	0.94(0.51–1.07)	0.041	0.26(0.21–1.24)	0.044*
**Ethnicity**						
Punjabi	2258(43.1%)	1,742(43.3%)	1.35(0.33–5.62)	0.365	1.72(0.47–6.98)	0.418
Sindhi	718(13.7%)	358(49.8%)	1.27(0.54–1.76)	0.178	1.21(0.23–1.88)	0.192
Balochi	597(11.4%)	216(36.1%)	0.87(0.21–1.29)	0.876	0.99(0.37–2.56)	0.717
Pashtun	1,366(26.1%)	1,165(85.2%)	0.26(0.17–1.86)	0.012	0.19(0.11–2.76)	0.010*
Other	298(5.7%)	198(66.4%)	1.03(0.43–1.87)	0.893	1.23(0.61–1.92)	0.896
**Marital Status**						
Single	3,891(74.3%)	2,798(71.9%)	0.62(0.12–0.78)	0.005	0.54(0.11–0.82)	0.001*
Married	1,346(25.7%)	881(65.4%)	1.30(0.72–2.38)	0.184	1.75(0.87–5.06)	0.261
**Specialty**						
Medicine/Allied	1,985(37.9%)	1,825(91.9%)	0.99(0.60–1.54)	0.001	0.83(0.51–1.26)	0.001*
Surgery/Allied	1,158(22.1%)	971(83.8)	0.76(0.53–1.39)	0.054	0.48(0.13–1.70)	0.091
Diagnostics	1,340(25.6%)	537(40%)	0.86(0.37–2.03)	0.365	1.04(0.53–2.04)	0.603
Other	754(14.4%)	346(45.8%)	1.34(0.93–2.15)	0.765	1.37(0.83–2.27)	0.518
**Type of medical facility**						
Tertiary care hospital	2682(51.2%)	2005(74.7%)	0.57(0.15–0.83)	0.734	0.72(0.45–0.96)	0.562
Primary care hospital	1598(30.5%)	1200(75%)	0.266(0.198–0.336)	0.547	0.273(0.211, 0.336)	0.235
Private hospital/clinic	957(18.2%)	474(49.5%)	0.257 (0.19–0.324)	0.126	0.250(0.154, 0.346)	0.611
**Designated work**						
Direct patient care provider	3,210(61.3%)	2,783(86.6%)	1.67(0.88–3.06)	0.001	1.39(0.80–2.41)	0.007*
No direct patient contact	2,027(38.7%)	896(44.2%)	0.81(0.57–1.32)	0.198	1.52(0.23–3.15)	0.216
**Education**						
Technical training	383(7.3%)	305(79.6%)	1.63(0.92–2.66)	0.614	2.21(1.02–4.59)	0.354
Bachelor’s degree	1,477(28.2%)	1,206(81.6%)	1.64(0.78–3.32)	0.001	1.15(0.44–3.97)	0.001*
Master’s degree	608(11.6%)	453(74.5%)	2.09(1.06–4.24)	0.043	2.01(1.06–4.00	0.072
Doctorate degree	545(10.4%)	211(38.7%)	1.35(0.44–3.36)	0.723	1.37(0.73–2.27)	0.658
**Medical conditions**						
None	2,776(53%)	1,809(65.1%)	1.47(0.76–2.81)	0.437	1.57(0.84–3.01)	0.481
DM I/II	378(7.2%)	309(81.7%)	2.12(0.63–3.33)	0.046	2.69(1.51–5.69)	0.059
Hypertension	576(11%)	427(74.1%)	1.30(0.67–2.17)	0.087	1.01(0.61–2.21)	0.065
Obesity	681(13%)	557(81.7%)	0.90(0.63–1.27)	0.092	1.18(0.56–2.48)	0.130
Smoking	351(6.7%)	254(72.3%)	0.70(0.47–1.02)	0.653	1.12(0.47–2.69)	0.376
Chronic respiratory condition	204(3.9%)	97(47.5%)	0.93(0.52–1.77)	0.093	1.01(0.23–4.45)	0.047*
Heart disease	120(2.3%)	102(85%)	1.23(0.99–1.69)	0.043	1.04(0.53–2.04)	0.021*
Renal failure	68(1.3%)	43(63.2%)	0.91(0.56–1.23)	0.974	1.37(0.83–2.27)	0.465
Cancer	83(1.6%)	81(97.5%)	1.43(1.05–1.92)	0.001	1.21(1.01–1.65)	0.004*
**Previous infection with COVID-19**	3,299(63%)	1,176(35.6%)	0.88(0.41–0.99)	0.001	0.43(0.27–1.08)	0.001*

Variables presented as n (%), adjusted for age, gender, and ethnicity.

*p-value <0.05. Diabetes mellitus (DM).

Acceptance rates of COVID-19 vaccination increased with increasing age. In the 18–30 age group, 64.6% of the respondents accepted the COVID-19 vaccine which increased to 71.2% in 31–40 years and 91.2% in 51–60 years. In >60 years 74.4% accepted for vaccination. Other factors predictive of vaccine acceptance were female gender and single relationship status. The female gender had a higher vaccine acceptance (80.7%) as well as those with a single relationship status (71.9%). A marked difference was seen in the vaccine acceptance among different ethnic groups. Pashtuns (85.2%) had the highest COVID-19 vaccine acceptance while Balochi’s had the lowest acceptance rate (36.1%). Vaccine acceptance varied among various specialties in healthcare. Those working in the specialty of medicine and allied (91.9%), in primary and tertiary healthcare settings (75% and 74.7%) had the highest vaccine acceptance and HCW’s with no direct patient contact had a high refusal rate (55.8%).

**Fig 2:**
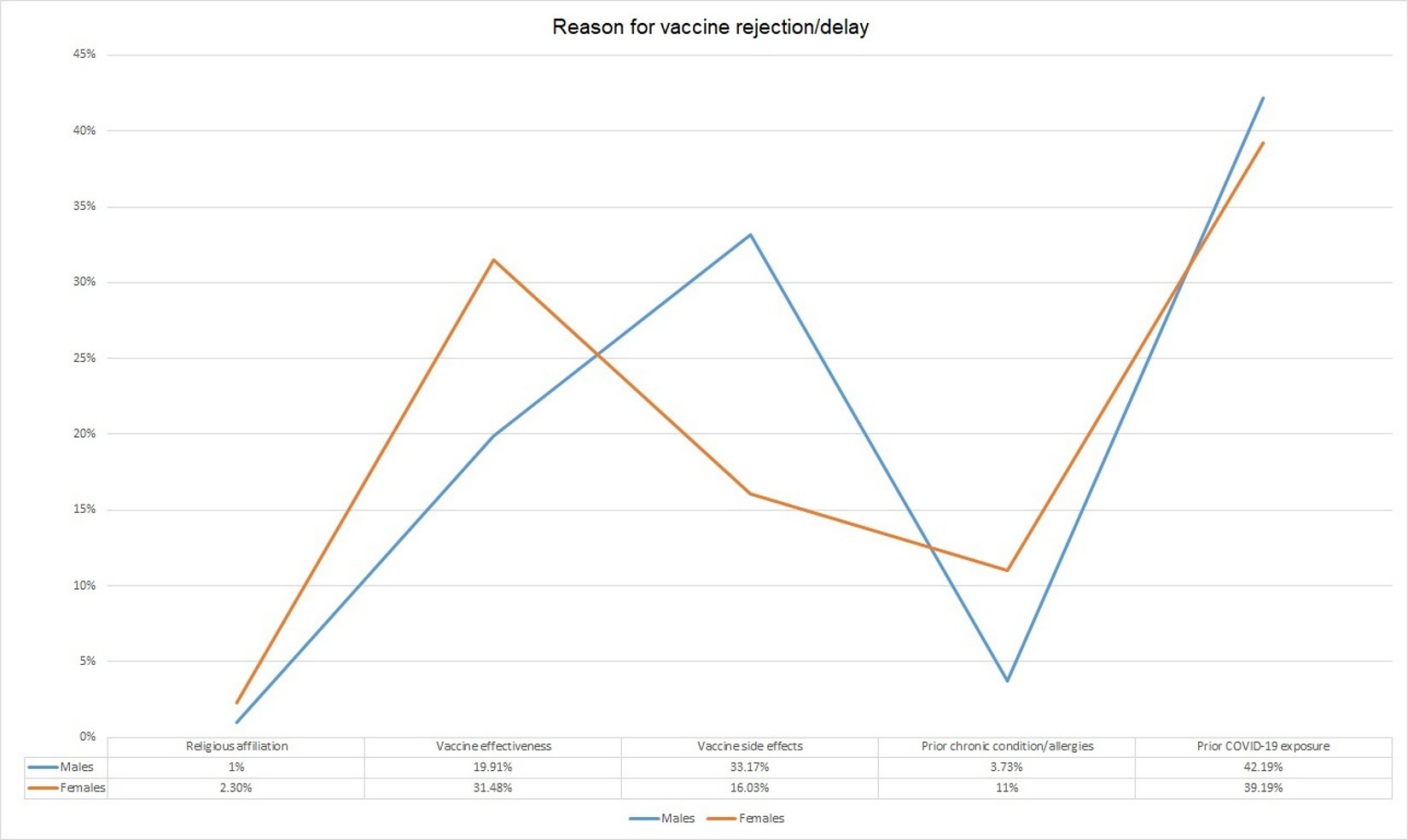
Views among genders regarding non-acceptance of vaccine.

**Fig 3:**
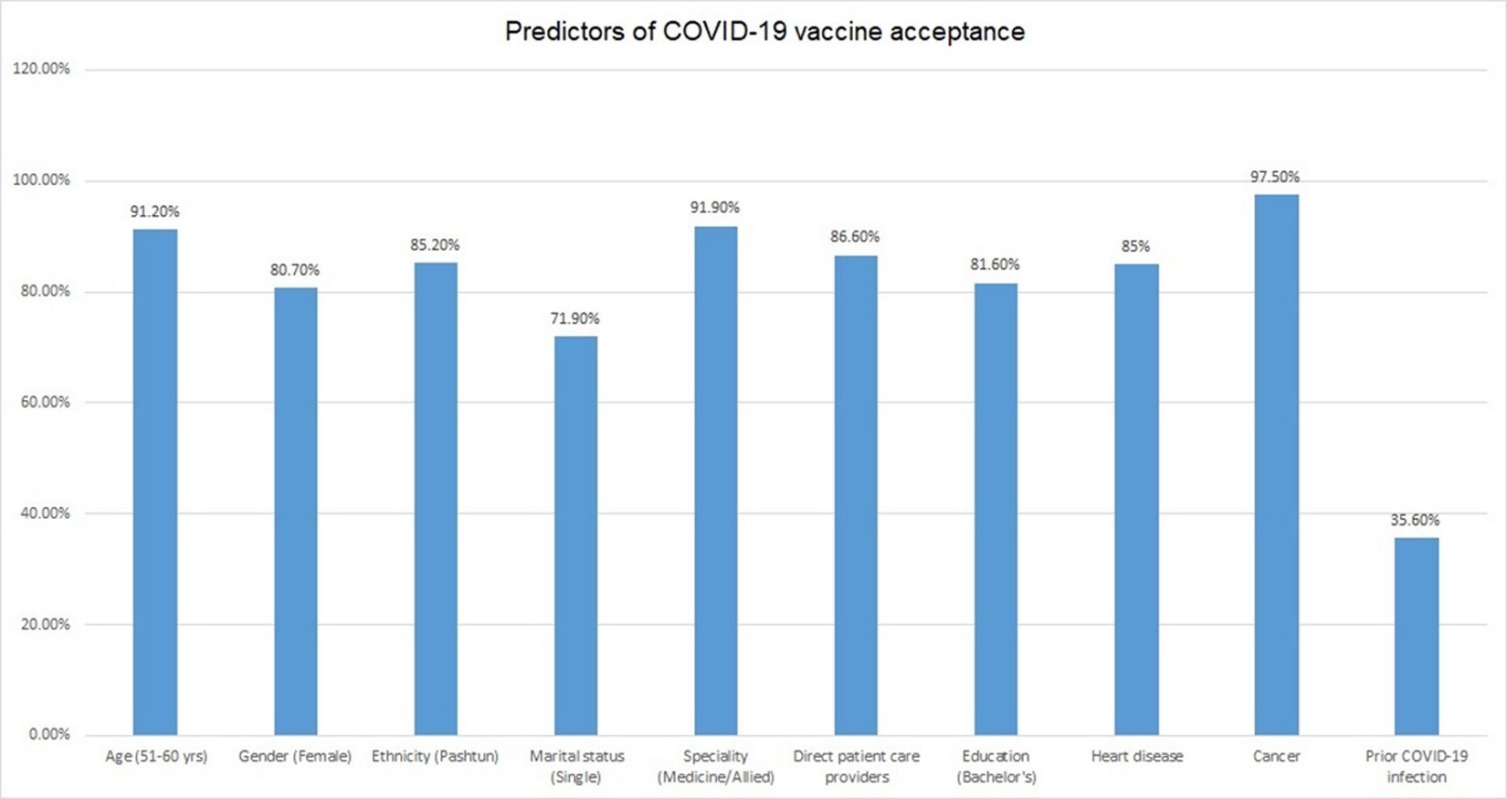
Logistic regression analysis demonstrating predictors for COVID-19 vaccine acceptance.

Among the two genders, there were different reasons for rejection of the COVID-19 vaccine (**Fig 2**). Females had religious concerns (2.3%) as compared to males (1%) and they were not convinced about the effectiveness of the vaccine (31.48%). The males not willing for the COVID-19 vaccine had prior COVID-19 infection (42.1%) and they were not sure about the side effects of the vaccine (33.1%). Logistic regression analysis demonstrated age between 51–60 years, female gender, Pashtuns, those working in the specialty of medicine and allied, taking direct care of COVID-19 patients, higher education, and prior OCVID-19 infection as the predictors for acceptance or rejection of COVID-19 vaccine (**Fig 3**).

## Discussion

The importance of healthcare workers in being strong advocates for eliminating vaccine hesitancy among vulnerable populations has previously been recognized by the WHO [10]. Recent evidence from research into this population subgroup shows that vaccine hesitancy in healthcare workers is often the result of lack of information regarding the vaccines, lack of confidence in communicating information about vaccines to parents and concerned family members, lack of trust in government authorities providing the vaccine and influence of social media posts on their decision making process [11–14]. Vaccine acceptability for COVID-19 among HCWs in South Asia is unknown which may become an impediment in national healthcare systems and create hurdles in vaccine uptake among the general population, prolonging return to normalization and delaying an end to the pandemic [11, 12, 14, 15].

In contrast to Pakistan’s difficult history in controlling Polio, due to vaccine reluctance in many of its endemic regions, such as the provinces of Khyber-Pakhtunkhwa and Balochistan [16–18], our study results seem to indicate that COVID-19 vaccination did not elicit such hesitancy prior to mass vaccination efforts by the government [9]. At present, Pakistan has been spared the worst of the pandemic’s toll on its healthcare system, mostly as a result of its demographic profile of having younger population and early adherence with mask and social distancing mandates, Pakistan’s cases and mortality have been lower than the developed countries. Other South Asian countries, having similar socioeconomic and demographic characteristics to Pakistan have fared worse in terms of COVID-19-associated morbidity and mortality [19, 20].

The incidence of COVID-19 in Pakistani HCWs, particularly in the population subset of our study, was consistent with other countries. This may indicate the need for more robust infection control practices in healthcare facilities on a global scale [20, 21]. It also reflected a reduced lack of risk perception, especially by male HCWs, who felt decreased need for COVID-19 vaccination in light of a previous infection. This is consistent among HCWs worldwide due to the limited data available about the severity of COVID-19 re-infection [22, 23]. and long-term health impairment even after recovery from COVID-19 [24, 25].

In our study, most of the HCWs accepted receiving a COVID-19 vaccine (70.2%), and only 5.2% rejected it completely. Approximately one-fourth of the respondents wanted more data on the vaccine before going forward with the vaccination process. Taken as a whole, our data suggests that COVID-19 vaccine acceptance among HCWs is higher as compared to general population worldwide [7, 26, 27]. Studies have presented a forecast model for spread of COVID-19 in Pakistan [28, 29]. however, these forecasts can be an underestimation if aggressive and timely vaccination drives are not administered through a sound government campaign.

Our study found female HCWs to be more accepting of vaccination, similar to other studies done in the region [11, 30]. However, a survey from France demonstrated a positive response for vaccine acceptance among majority of the participants (71.2%) and outright refusal was associated with female gender, age, lower educational status, and no report of chronic disease [26]. In contrast to these results, female gender was more likely to get vaccinated in our study and percentage of vaccine acceptance increased with increasing age. Furthermore, we found HCWs in direct patient care to be more accepting of vaccination against COVID-19 compared to those HCWs who were involved in indirect patient care. Acceptability by age in our study was higher among the 51–60 years’ group, which is similar to other studies where increasing age and education are both positive factors in vaccine acceptance [11, 15, 31].

The variation in hesitancy by ethnicity showed a marked difference from previously performed studies in Pakistan, where Khyber Pakhtunkhwa has often been singled as being more vaccine resistant than the rest of the country [32]. However, our study showed that HCWs of Pashtun ethnicity were surprisingly more likely to get vaccinated compared to their counterparts from other provinces of Pakistan. To date, few studies have investigated acceptance of COVID-19 vaccines specifically among HCWs from various ethnicities in the same country. This is also indicative of the various healthcare disparities that exist in a developing country like Pakistan, where minority groups may be at the lower end of health resource allocation and utilization by the government, worsening distrust of vaccines and healthcare workers in these regions [18, 33].

Being a predominantly Muslim country, religion has often been a strong factor in rejecting vaccination for various vaccine preventable diseases in Pakistan, with many citing the contents of the vaccines to be non-compliant to Sharia law and therefore religiously unacceptable to them [7, 11, 34]. These findings were also reflected in our study, even among highly educated HCWs, particularly those who were female. However, recent public statements by major Islamic organizations have outlined that no such incompatibility exits [17].

Although, not directly addressed in our study, one important aspect of COVID-19 vaccine hesitancy in Pakistan, was the impact of social media as a source of information for HCWs during this pandemic. Social media posts have been implicated in similar studies carried out in Muslim majority Middle Eastern countries [11, 35]. Combatting this ‘infodemic’ with timely, evidence based communication is necessary to ensure that misinformation does not hamper national vaccination efforts [36].

A major strength of our study was the robust sample size of HCWs who responded to the questionnaire and this survey represents a diverse group of individuals working as health care providers. However, there were some limitations to this study. A snowball sampling method could have created a selection and social desirability bias among HCWs. Furthermore, English questionnaire can produce a selection bias towards English-literate HCWs, particularly those active on social media. Despite these limitations, an overall positive response to vaccine acceptability is a positive sign towards attaining herd immunity worldwide, and increasing information and health communication from healthcare workers to the general population will decrease hesitancy towards COVID-19 vaccines.

## Conclusion

In conclusion, this survey suggests that early on in a vaccination drive, majority of the HCWs in Pakistan are willing to be vaccinated and only a small number of participants would actually reject being vaccinated. Overall, we gathered positive response towards COVID-19 vaccines but specific concerns regarding effectiveness and side effects were prevalent. Differences in vaccine acceptance were seen in various health care specialties, age groups, and ethnicity. Acceptance of COVID-19 vaccine in Pakistan is influenced by the evidence of vaccine effectiveness and while the acceptability among HCWs in Pakistan is higher than other surveys, a clear communication by the government, using the experience of HCWs as trusted sources of medical information, is needed to ensure the success of a national vaccination strategy.

## References

[1] World Health Organization. Novel Coronavirus (2019-nCoV) SITUATION REPORT—1 21 JANUARY 2020 [Internet]. World Health Organization; 2020 [cited 2021 Feb 16]. Available from: https://www.who.int/docs/default-source/coronaviruse/situation-reports/20200121-sitrep-1-2019-ncov.pdf?sfvrsn=20a99c10_4

[2] Huang C, Wang Y, Li X, Ren L, Zhao J, Hu Y, et al. Clinical features of patients infected with 2019 novel coronavirus in Wuhan, China. The Lancet [Internet] 2020 [cited 2021 Mar 29];395(10223):497–506. Available from: https://linkinghub.elsevier.com/retrieve/pii/S0140673620301835 10.1016/S0140-6736(20)30183-5 PMC7159299 31986264

[3] Rodriguez-Morales AJ, Cardona-Ospina JA, Gutiérrez-Ocampo E, Villamizar-Peña R, Holguin-Rivera Y, Escalera-Antezana JP, et al. Clinical, laboratory and imaging features of COVID-19: A systematic review and meta-analysis. Travel Med Infect Dis 2020;34:101623. doi: 10.1016/j.tmaid.2020.101623 32179124 PMC7102608

[4] World Health Organization. WHO Coronavirus Disease (COVID-19) Dashboard | WHO Coronavirus Disease (COVID-19) Dashboard [Internet]. 2020 [cited 2021 Feb 16];Available from: https://covid19.who.int/

[5] National Command Operation. National Command Operation Center [Internet]. 2020 [cited 2021 Feb 16];Available from: https://ncoc.gov.pk/sitrep.php

[6] Tabah A, Ramanan M, Laupland KB, Buetti N, Cortegiani A, Mellinghoff J, et al. Personal protective equipment and intensive care unit healthcare worker safety in the COVID-19 era (PPE-SAFE): An international survey. J Crit Care 2020;59:70–5. doi: 10.1016/j.jcrc.2020.06.005 32570052 PMC7293450

[7] Saied SM, Saied EM, Kabbash IA, Abdo SAE. Vaccine hesitancy: Beliefs and barriers associated with COVID‐19 vaccination among Egyptian medical students. J Med Virol [Internet] 2021 [cited 2021 Apr 8];Available from: https://www.ncbi.nlm.nih.gov/pmc/articles/PMC8013865/ doi: 10.1002/jmv.26910 33644891 PMC8013865

[8] Lucia VC, Kelekar A, Afonso NM. COVID-19 vaccine hesitancy among medical students. J Public Health (Oxf) 2020;fdaa230. doi: 10.1093/pubmed/fdaa230 33367857 PMC7799040

[9] Web Correspondant. Phase II: Pakistan to start vaccinating citizens over 65 years on receiving 2.8m doses around March 2, says SAPM—DAWN.COM [Internet]. Dawn2021 [cited 2021 Feb 20];Available from: https://www.dawn.com/news/1608067

[10] Butler R, MacDonald NE. Diagnosing the determinants of vaccine hesitancy in specific subgroups: The Guide to Tailoring Immunization Programmes (TIP). Vaccine [Internet] 2015 [cited 2021 Feb 17];33(34):4176–9. Available from: https://linkinghub.elsevier.com/retrieve/pii/S0264410X15005022 doi: 10.1016/j.vaccine.2015.04.038 25896376

[11] Elbarazi I, Al-Hamad S, Alfalasi S, Aldhaheri R, Dubé E, Alsuwaidi AR. Exploring vaccine hesitancy among healthcare providers in the United Arab Emirates: a qualitative study. Hum Vaccin Immunother 2020;1–8. doi: 10.1080/21645515.2020.1855953 33369524 PMC8189094

[12] Harapan H, Wagner AL, Yufika A, Winardi W, Anwar S, Gan AK, et al. Acceptance of a COVID-19 Vaccine in Southeast Asia: A Cross-Sectional Study in Indonesia. Front Public Health [Internet] 2020 [cited 2021 Feb 13];8:381. Available from: https://www.frontiersin.org/article/10.3389/fpubh.2020.00381/full 32760691 10.3389/fpubh.2020.00381 PMC7372105

[13] Kabamba Nzaji M, Kabamba Ngombe L, Ngoie Mwamba G, Banza Ndala DB, Mbidi Miema J, Luhata Lungoyo C, et al. Acceptability of Vaccination Against COVID-19 Among Healthcare Workers in the Democratic Republic of the Congo. POR [Internet] 2020 [cited 2021 Feb 17];Volume 11:103–9. Available from: https://www.dovepress.com/acceptability-of-vaccination-against-covid-19-among-healthcare-workers-peer-reviewed-article-POR doi: 10.2147/POR.S271096 33154695 PMC7605960

[14] Abbas Q, Mangrio F, Kumar S. Myths, beliefs, and conspiracies about COVID-19 Vaccines in Sindh, Pakistan: An online cross-sectional survey. Authorea [Internet] 2021 [cited 2021 Apr 5];Available from: https://www.authorea.com/users/400163/articles/512518-myths-beliefs-and-conspiracies-about-covid-19-vaccines-in-sindh-pakistan-an-online-cross-sectional-survey?commit=bf5ff20f4aab9c35890625a779175d881bbb1901

[15] Sallam M. COVID-19 Vaccine Hesitancy Worldwide: A Concise Systematic Review of Vaccine Acceptance Rates. Vaccines [Internet] 2021 [cited 2021 Feb 17];9(2):160. Available from: https://www.mdpi.com/2076-393X/9/2/160 doi: 10.3390/vaccines9020160 33669441 PMC7920465

[16] Ali M, Ahmad N, Khan H, Ali S, Akbar F, Hussain Z. Polio vaccination controversy in Pakistan. The Lancet [Internet] 2019 [cited 2021 Mar 30];394(10202):915–6. Available from: https://www.thelancet.com/journals/lancet/article/PIIS0140-6736(19)32101-4/abstract doi: 10.1016/S0140-6736(19)32101-4 31526731

[17] Babakhel Z. Islamic Advisory Group (IAG) Appreciates Pakistan’s Resolve to End Polio from the Country [Internet]. Pakistan Polio Eradication Programme 2020 [cited 2020 Dec 14];Available from: https://www.endpolio.com.pk/media-room/media-releases/618-islamic-advisory-group-iag-appreciates-pakistan-s-resolve-to-end-polio-from-the-country

[18] Panezai S, Ahmad MM, Saqib SE. Factors affecting access to primary health care services in Pakistan: a gender-based analysis. Development in Practice [Internet] 2017 [cited 2021 Jun 25];27(6):813–27. Available from: https://www.tandfonline.com/doi/full/10.1080/09614524.2017.1344188

[19] Rueda ST, Sweeney S, Bozzani F, Vassall A. The health sector cost of different policy responses to COVID-19 in low-and middle-income countries. medRxiv 2020;

[20] Nawaz A, Su X, Barkat MQ, Asghar S, Asad A, Basit F, et al. Epidemic Spread and Its Management Through Governance and Leadership Response Influencing the Arising Challenges Around COVID-19 in Pakistan—A Lesson Learnt for Low Income Countries With Limited Resource. Front Public Health [Internet] 2020 [cited 2021 Feb 20];8. Available from: https://www.ncbi.nlm.nih.gov/pmc/articles/PMC7758222/ doi: 10.3389/fpubh.2020.573431 33363079 PMC7758222

[21] Abid K, Bari YA, Younas M, Tahir Javaid S, Imran A. Progress of COVID-19 Epidemic in Pakistan. Asia Pac J Public Health [Internet] 2020 [cited 2020 Oct 2];32(4):154–6. Available from: http://journals.sagepub.com/doi/10.1177/1010539520927259 10.1177/1010539520927259 PMC7240311 32429679

[22] Cohen JI, Burbelo PD. Reinfection with SARS-CoV-2: Implications for Vaccines. Clin Infect Dis 2020; doi: 10.1093/cid/ciaa1866 33338197 PMC7799323

[23] Hanif M, Haider MA, Ali MJ, Naz S, Sundas FNU. Reinfection of COVID-19 in Pakistan: A First Case Report. Cureus [Internet] 2020 [cited 2021 Mar 31];12(10). Available from: https://www.cureus.com/articles/40689-reinfection-of-covid-19-in-pakistan-a-first-case-report doi: 10.7759/cureus.11176 33262913 PMC7689968

[24] Dasgupta A, Kalhan A, Kalra S. Long term complications and rehabilitation of COVID-19 patients. J Pak Med Assoc [Internet] 2020 [cited 2020 Sep 26];(0):1. Available from: https://www.ejmanager.com/fulltextpdf.php?mno=105926 32515393 10.5455/JPMA.32

[25] Lopez-Leon S, Wegman-Ostrosky T, Perelman C, Sepulveda R, Rebolledo PA, Cuapio A, et al. More than 50 Long-term effects of COVID-19: a systematic review and meta-analysis. medRxiv [Internet] 2021 [cited 2021 Feb 21];Available from: https://www.ncbi.nlm.nih.gov/pmc/articles/PMC7852236/10.1038/s41598-021-95565-8 PMC8352980 34373540

[26] Schwarzinger M, Watson V, Arwidson P, Alla F, Luchini S. COVID-19 vaccine hesitancy in a representative working-age population in France: a survey experiment based on vaccine characteristics. The Lancet Public Health [Internet] 2021 [cited 2021 Mar 29];6(4):e210–21. Available from: https://www.thelancet.com/journals/lanpub/article/PIIS2468-2667(21)00012-8/abstract doi: 10.1016/S2468-2667(21)00012-8 33556325 PMC7864787

[27] Verger P, Fressard L, Collange F, Gautier A, Jestin C, Launay O, et al. Vaccine Hesitancy Among General Practitioners and Its Determinants During Controversies: A National Cross-sectional Survey in France. EBioMedicine [Internet] 2015 [cited 2021 Apr 5];2(8):891–7. Available from: https://www.sciencedirect.com/science/article/pii/S2352396415300475 doi: 10.1016/j.ebiom.2015.06.018 26425696 PMC4563133

[28] Ali M, Khan DM, Aamir M, Khalil U, Khan Z. Forecasting COVID-19 in Pakistan. PLoS One 2020;15(11):e0242762. doi: 10.1371/journal.pone.0242762 33253248 PMC7703963

[29] Khan F, Saeed A, Ali S. Modelling and forecasting of new cases, deaths and recover cases of COVID-19 by using Vector Autoregressive model in Pakistan. Chaos, Solitons & Fractals [Internet] 2020 [cited 2020 Oct 13];140:110189. Available from: http://www.sciencedirect.com/science/article/pii/S0960077920305853 doi: 10.1016/j.chaos.2020.110189 32834659 PMC7405884

[30] Kuter BJ, Browne S, Momplaisir FM, Feemster KA, Shen AK, Green-McKenzie J, et al. Perspectives on the receipt of a COVID-19 vaccine: A survey of employees in two large hospitals in Philadelphia. Vaccine [Internet] 2021 [cited 2021 Apr 8];39(12):1693–700. Available from: https://www.ncbi.nlm.nih.gov/pmc/articles/PMC7885691/ doi: 10.1016/j.vaccine.2021.02.029 33632563 PMC7885691

[31] Karafillakis E, Dinca I, Apfel F, Cecconi S, Wűrz A, Takacs J, et al. Vaccine hesitancy among healthcare workers in Europe: A qualitative study. Vaccine 2016;34(41):5013–20. doi: 10.1016/j.vaccine.2016.08.029 27576074

[32] Shah SFA, Ginossar T, Weiss D. “This is a Pakhtun disease”: Pakhtun health journalists’ perceptions of the barriers and facilitators to polio vaccine acceptance among the high-risk Pakhtun community in Pakistan. Vaccine 2019;37(28):3694–703. doi: 10.1016/j.vaccine.2019.05.029 31155417

[33] Jennings L, Gagliardi L. Influence of mhealth interventions on gender relations in developing countries: a systematic literature review. International Journal for Equity in Health [Internet] 2013 [cited 2021 Mar 15];12(1):85. Available from: doi: 10.1186/1475-9276-12-85 24131553 PMC4015705

[34] Robertson E, Reeve KS, Niedzwiedz CL, Moore J, Blake M, Green M, et al. Predictors of COVID-19 vaccine hesitancy in the UK household longitudinal study. Brain Behav Immun [Internet] 2021 [cited 2021 Jun 25];94:41–50. Available from: https://www.ncbi.nlm.nih.gov/pmc/articles/PMC7946541/ doi: 10.1016/j.bbi.2021.03.008 33713824 PMC7946541

[35] Sallam M, Dababseh D, Eid H, Al-Mahzoum K, Al-Haidar A, Taim D, et al. High Rates of COVID-19 Vaccine Hesitancy and Its Association with Conspiracy Beliefs: A Study in Jordan and Kuwait among Other Arab Countries. Vaccines (Basel) [Internet] 2021 [cited 2021 Feb 21];9(1). Available from: https://www.ncbi.nlm.nih.gov/pmc/articles/PMC7826844/ doi: 10.3390/vaccines9010042 33445581 PMC7826844

[36] World Health Organization. Infodemic [Internet]. Infodemic2020 [cited 2021 Feb 23];Available from: https://www.who.int/health-topics/infodemic

